# Tetracyclic homoisoflavanoid (+)-brazilin: a natural product inhibits c-di-AMP-producing enzyme and *Streptococcus mutans* biofilms

**DOI:** 10.1128/spectrum.02418-23

**Published:** 2024-04-09

**Authors:** Edwin M. Rojas, Hua Zhang, Sadanandan E. Velu, Hui Wu

**Affiliations:** 1 School of Dentistry, University of Alabama at Birmingham, Birmingham, Alabama, USA; 2 Department of Chemistry, University of Alabama at Birmingham, Birmingham, Alabama, USA; 3 Division of Biomaterial & Biomedical Sciences, School of Dentistry, Oregon Health & Science University, Portland, Oregon, USA; University of Florida College of Dentistry, Gainesville, Florida, USA

**Keywords:** dental caries, *Streptococcus mutans*, cyclic di-AMP, diadenylate cyclase, (+)-brazilin, natural products, biofilm inhibition

## Abstract

The tenacious biofilms formed by *Streptococcus mutans* are resistant to conventional antibiotics and current treatments. There is a growing need for novel therapeutics that selectively inhibit *S. mutans* biofilms while preserving the normal oral microenvironment. Previous studies have shown that increased levels of cyclic di-AMP, an important secondary messenger synthesized by diadenylate cyclase (DAC), favored biofilm formation in *S. mutans*. Thus, targeting *S. mutans* DAC is a novel strategy to inhibit *S. mutans* biofilms. We screened a small NCI library of natural products using a fluorescence detection assay. (+)-Brazilin, a tetracyclic homoisoflavanoid found in the heartwood of *Caesalpinia sappan*, was identified as one of the 11 “hits,” with the greatest reduction (>99%) in fluorescence at 100 µM. The *sm*DAC inhibitory profiles of the 11 “hits” established by a quantitative high-performance liquid chromatography assay revealed that (+)-brazilin had the most enzymatic inhibitory activity (87% at 100 µM) and was further studied to determine its half maximal inhibitory concentration (IC_50_ = 25.1 ± 0.98 µM). (+)-Brazilin non-competitively inhibits *sm*DAC’s enzymatic activity (*K*_*i*_ = 140.0 ± 27.13 µM), as determined by a steady-state Michaelis-Menten kinetics assay. In addition, (+)-brazilin’s binding profile with *sm*DAC (*K*_*d*_ = 11.87 µM) was illustrated by a tyrosine intrinsic fluorescence quenching assay. Furthermore, at low micromolar concentrations, (+)-brazilin selectively inhibited the biofilm of *S. mutans* (IC_50_ = 21.0 ± 0.60 µM) and other oral bacteria. *S. mutans* biofilms were inhibited by a factor of 10^5^ in colony-forming units when treated with 50 µM (+)-brazilin. In addition, a significant dose-dependent reduction in extracellular DNA and glucan levels was evident by fluorescence microscopy imaging of *S. mutans* biofilms exposed to different concentrations of (+)-brazilin. Furthermore, colonization of *S. mutans* on a representative model of enamel using suspended hydroxyapatite discs showed a >90% reduction with 50 µM (+)-brazilin. In summary, we have identified a drug-like natural product inhibitor of *S. mutans* biofilm that not only binds to *sm*DAC but can also inhibit the function of *sm*DAC. (+)-Brazilin could be a good candidate for further development as a potent therapeutic for the prevention and treatment of dental caries.

**IMPORTANCE:**

This study represents a significant advancement in our understanding of potential therapeutic options for combating cariogenic biofilms produced by *Streptococcus mutans*. The research delves into the use of (+)-brazilin, a natural product, as a potent inhibitor of *Streptococcus mutans*’ diadenylate cyclase (*sm*DAC), an enzyme crucial in the formation of biofilms. The study establishes (+)-brazilin as a non-competitive inhibitor of *sm*DAC while providing initial insights into its binding mechanism. What makes this finding even more promising is that (+)-brazilin does not limit its inhibitory effects to *S. mutans* alone. Instead, it demonstrates efficacy in hindering biofilms in other oral bacteria as well. The broader spectrum of anti-biofilm activity suggests that (+)-brazilin could potentially serve as a versatile tool in a natural product-based treatment for combating a range of conditions caused by resilient biofilms.

## INTRODUCTION

Dental caries is a widespread disease caused by the demineralization of tooth enamel by acid produced by oral microbes that reside in dental plaque (1). Dental plaque consists of more than 700 different bacterial species living in complex bacterial communities called biofilms that mediate health and disease (2). Although several oral bacteria are associated with different forms of dental caries, *Streptococcus mutans* has been implicated as the major etiological agent in the initiation and development of this disease, especially early childhood caries (3–5). As biofilms mature and become difficult to disrupt, a sugar-rich environment within the complex biofilm matrix stimulates the metabolism of carbohydrates and the production of lactic acid, consequently eroding and demineralizing the enamel and leading to carious infection (6). The tenacious biofilms formed by *S. mutans* are resistant to conventional antibiotics and currently marketed dental caries treatments (7–11).

**Fig 1:**
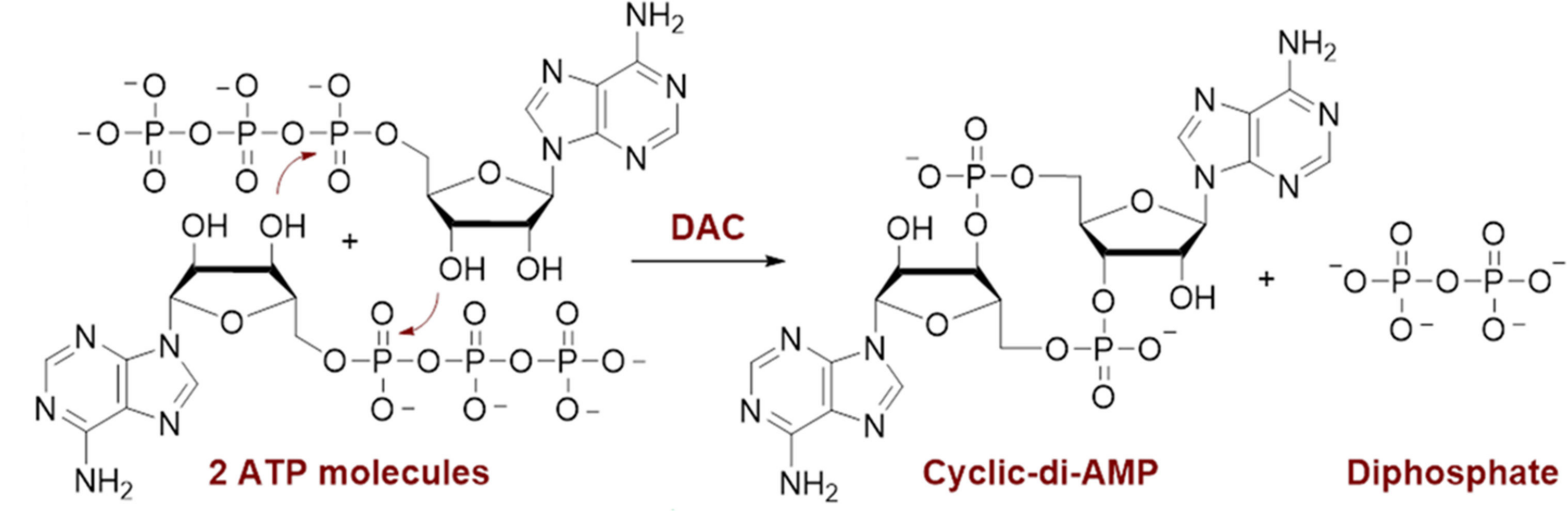
Diadenylate cyclase enzyme converts two ATP molecules into c-di-AMP.

Recent studies have shown that increased levels of cyclic di-AMP (c-di-AMP), an important secondary messenger in *S. mutans*, favored biofilm formation (12). c-di-AMP, a cyclic dinucleotide, regulates many processes in Gram-positive bacteria, such as potassium uptake, fatty acid synthesis, and cell wall homeostasis (13). c-di-AMP is synthesized from two ATP molecules by diadenylate cyclase (DAC) enzymes (Fig. 1). To date, only DACs have been shown to synthesize c-di-AMP, while other families of cyclases have evolved to specifically synthesize other dinucleotides, such as cyclic di-GMP (14). There are five classes of DAC enzymes: CdaA, CdaS, CdaR, CdaM, and DisA, all having a conserved catalytic domain (15). *sm*DAC belongs to the CdaA (DacA) family of DAC enzymes (16). A proposed mechanism by which c-di-AMP induces biofilm formation in *S. mutans* involves the binding of a c-di-AMP-binding protein (CabPA) to a transcriptional factor, VicR, known for upregulating the expression of *gtfB* (12). The gene *gtfB*, which codes for glucosyltransferase B (GtfB), has been shown to be an essential virulence factor of mutans streptococci associated with the development of cariogenic biofilms (17). GtfB is an extracellular enzyme that hydrolyzes dietary sucrose into fructose and glucose (18). GtfB then polymerizes glucose units into adhesive insoluble glucan chains, which make up the bulk of extracellular polysaccharide matrix in biofilms (18). In addition, c-di-AMP synthesized by *sm*DAC regulates other cellular processes important for bacterial stress responses. Therefore, the inhibition of *sm*DAC is a viable upstream strategy to inhibit *S. mutans* biofilms formed by the sucrose-dependent pathway.

To the best of our knowledge, there are no known inhibitors of *sm*DAC reported in the literature. However, there are several reports of screening and characterization methods to identify small-molecule inhibitors of DNA integrity scanning protein A (DisA) and CdaA enzymes in other microorganisms (19). These DisA inhibitors include bromophenol thiohydantoin, a non-competitive inhibitor (IC_50_ = 56 µM) (20); cordycepin triphosphate (3′-deoxyATP) (IC_50_ = 3 µM) (21); tannic acid (IC_50_ = 3.4 µM); theaflavin-3′-gallate (IC_50_ = 23.6 µM); theaflavin-3,3′-digallate (IC_50_ = 8.5 µM) (22); and suramin, an antiparasitic drug (IC_50_ = 2.3 µM) (23). Recently, 2,2′-dihydroxy-1,1′-dinapthyldisulfide (IPA-3) (IC_50_ = 38.22 µM) was found to inhibit *Streptococcus suis* CdaA and possess antimicrobial activities toward a variety of bacterial species (24). Although IPA-3 has an interesting drug-like scaffold, it lacks selectivity, has low solubility, and has shown cytotoxic effects on human cell lines (24, 25). Since there are reported DAC inhibitors, we decided to evaluate some of them and perform our own screening using fluorescence and high-performance liquid chromatography (HPLC) assays, which we have either modified or developed, to identify inhibitors of *sm*DAC.

Natural products are an important source for lead compounds due to their high structural rigidity, large number of sp^3^ atoms, structural diversity, and lower octanol-water partition coefficients, some of many factors that aid in the bioinspiration of next-generation drug candidates (26–29). In this study, we screened a small natural product library of 20 polyphenol compounds (obtained from the NCI) to identify “hits,” which inhibit c-di-AMP production using the coralyne fluorescence assay. From the 11 “hits” obtained from this assay, (+)-brazilin, a tetracyclic homoisoflavanoid found in the heartwood of *Caesalpinia sappan* also possessing anti-inflammatory, anti-allergic, anti-oxidant, anti-bacterial, anti-platelet, anti-cancer, and neuroprotective properties (30–42), showed the most reduction in fluorescence and was further evaluated using an HPLC assay (19, 20). We adopted an intrinsic tyrosine fluorescence assay to verify if (+)-brazilin binds to *sm*DAC. (+)-Brazilin has been previously reported as a biofilm inhibitor of *S. mutans* when isolated from an extract of *Caesalpinia sappan*; however, its potential target or mechanism of action has not been studied (43). We have tailored microbiological and fluorescence microscopy assays to further glean on (+)-brazilin’s effect on *S. mutans* biofilms by assessing glucan production and the presence of extracellular DNA (eDNA) (44–46). We have also employed a hydroxyapatite disc assay as a representative model biofilms on lateral surfaces of tooth structure (47–49). (+)-Brazilin’s effect on oral commensal streptococci was also investigated. In summary, (+)-brazilin is a new inhibitor of *S. mutans* biofilm*,* which can bind to and inhibit *sm*DAC.

## RESULTS

### Mn^2+^ cofactor is required for smDAC’s enzymatic activity

We screened several metal chlorides (MnCl_2_, MgCl_2_, CoCl_2_, and CaCl_2_) to determine which metal co-factor is optimal for *sm*DAC’s enzymatic activity. Treatment with MnCl_2_ showed optimal enzymatic activity (81% conversion), and some enzymatic activity was observed with CoCl_2_ (23% conversion), suggesting that *sm*DAC converts ATP into c-di-AMP selectively in the presence of Mn^2+^ or Co^2+^ *in vitro* (Fig. S2). No c-di-AMP was detected for enzymatic reactions treated with CaCl_2_, MgCl_2_, or without metal co-factor. Interestingly, reactions with 10 mM MnCl_2_ and 10% dimethyl sulfoxide (DMSO) (81% conversion) exhibited enzymatic activity comparable to reactions without DMSO (77% conversion), meaning that *sm*DAC is stable and enzymatically active in reaction buffer containing 10% DMSO. This finding facilitated the preparation and testing of screening compounds in DMSO. We also determined that the optimal Mn^2+^ concentration ranged from 5 to 10 mM for *sm*DAC’s enzymatic activity and noticed a gradual decline in activity at concentrations higher than 10 mM Mn^2+^ (Fig. S3).

### (+)-Brazilin, a natural product identified as a potential *sm*DAC inhibitor from the coralyne assay

**Fig 2:**
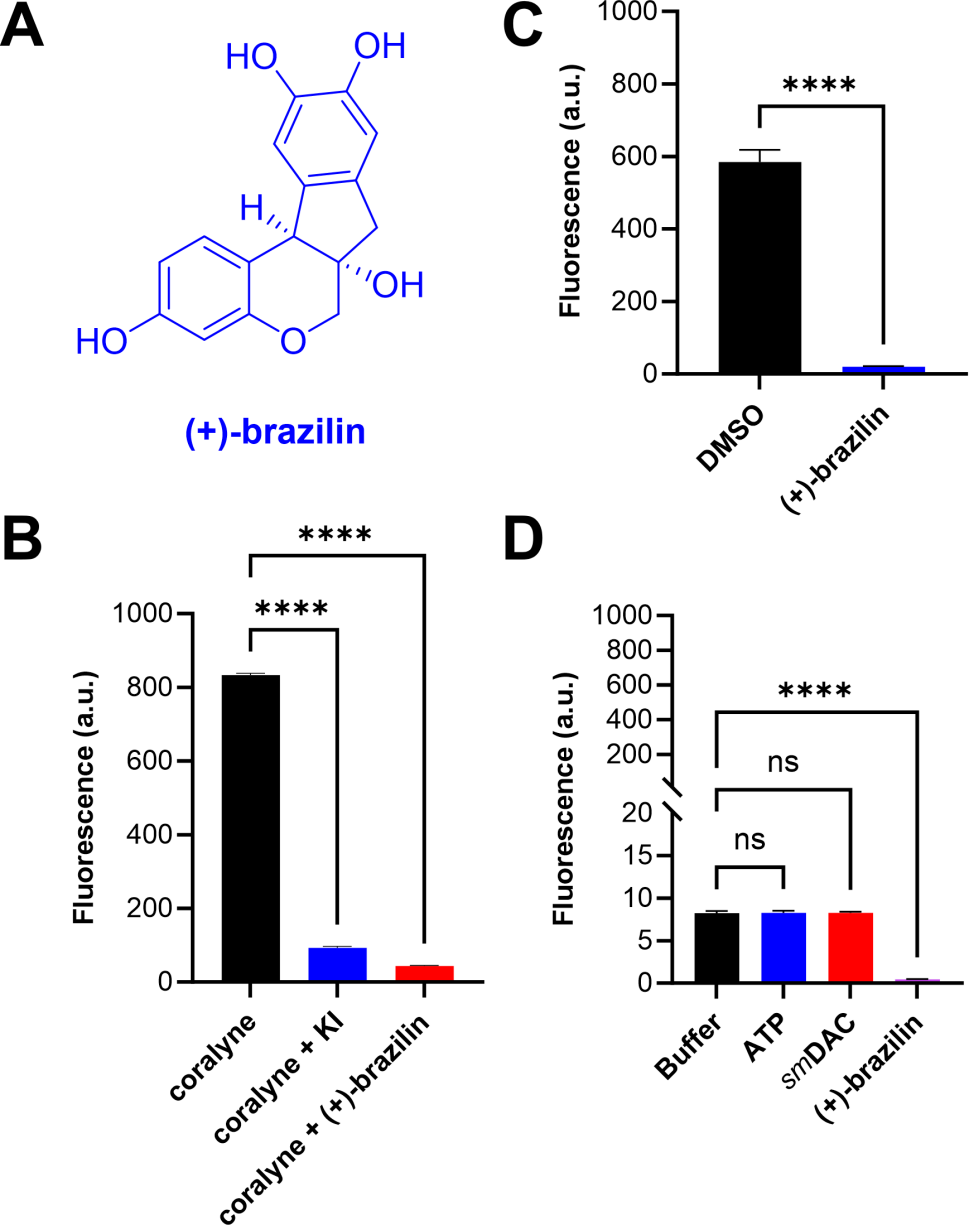
Evaluation of (+)-brazilin obtained from the coralyne assay. (A) Chemical structure of (+)-brazilin. (B) Quenching effect showing that (+)-brazilin and KI both quench the fluorescence of coralyne in the absence of *sm*DAC and ATP. Fluorescence (a.u.) was recorded at 475 nm after 4 h for 10 µM coralyne in reaction buffer, 10 µM coralyne + 10 mM KI in reaction buffer, and 10 µM coralyne + 100 µM (+)-brazilin in reaction buffer. (C) Fluorescence intensity (a.u.) at 475 nm after 4 h from *sm*DAC reactions in reaction buffer (containing 100 µM ATP, 5 µM *sm*DAC, 10 µM coralyne, and 10 mM KI) in the presence of 100 µM (+)-brazilin compared to 10% DMSO negative control. Reactions were performed in triplicate. Error bars denote the standard error of the mean (SEM). ****P value < 0.0001. Statistical analysis was performed by Student’s *t*-test using GraphPad Prism 10.0.3. Reactions were performed in triplicate. Error bars denote the SEM. ****P value < 0.0001. Statistical analysis was performed by one-way ANOVA and Dunnett’s multiple comparisons test using GraphPad Prism 10.0.3. (D) Fluorescence intensity (a.u.) of individual substrates at 475 nm after 4 h for 10% DMSO in reaction buffer, 100 µM ATP in reaction buffer, 5 µM *sm*DAC in reaction buffer, and 100 µM (+)-brazilin in reaction buffer. Reactions were performed in triplicate. Error bars denote the SEM. ****P value < 0.0001 and ns, not significant. Statistical analysis was performed by one-way ANOVA and Dunnett’s multiple comparisons test using GraphPad Prism 10.0.3.

We screened an NCI library of 20 natural products to obtain “hits” with the potential to inhibit c-di-AMP production. From the small library of 20 natural products, 14 compounds (100 µM) showed >75% reduction in fluorescence from the coralyne fluorescence assay (Fig. S4). Out of the 14, 11 were further validated using HPLC due to insufficient quantities of NSC numbers 85474, 61640, and 54304. NSC 8661, known as (+)-brazilin (Fig. 2A), showed >99% reduction in fluorescence, closely followed by NSC numbers 85474, 93087, 123262, 30665, and 113490, with >95% reduction in fluorescence (Fig. S4). (+)-Brazilin did not show an increase in fluorescence intensity at 100 µM compared to the negative control reaction, which showed the expected increase in fluorescence intensity as c-di-AMP forms a complex with coralyne over time (Fig. 2B). However, 100 µM (+)-brazilin quenched the fluorescence of coralyne (Fig. 2C). This finding suggested that a quantitative assay, such as HPLC, was required to confirm its inhibitory profile against *sm*DAC. Moreover, different ingredients such as 100 µM ATP, 5 µM *sm*DAC, 100 µM (+)-brazilin, and 10% DMSO in buffer were all examined to consider if they independently contribute to the fluorescence intensity over time (Fig. 2D). None showed significant fluorescence intensity levels, further confirming that fluorescence intensity from the enzymatic reaction was exclusively due to the increased concentration of the c-di-AMP-coralyne complex over time.

### (+)-Brazilin inhibits c-di-AMP production the most from the 11 “hits,” detected by HPLC

**Fig 3:**
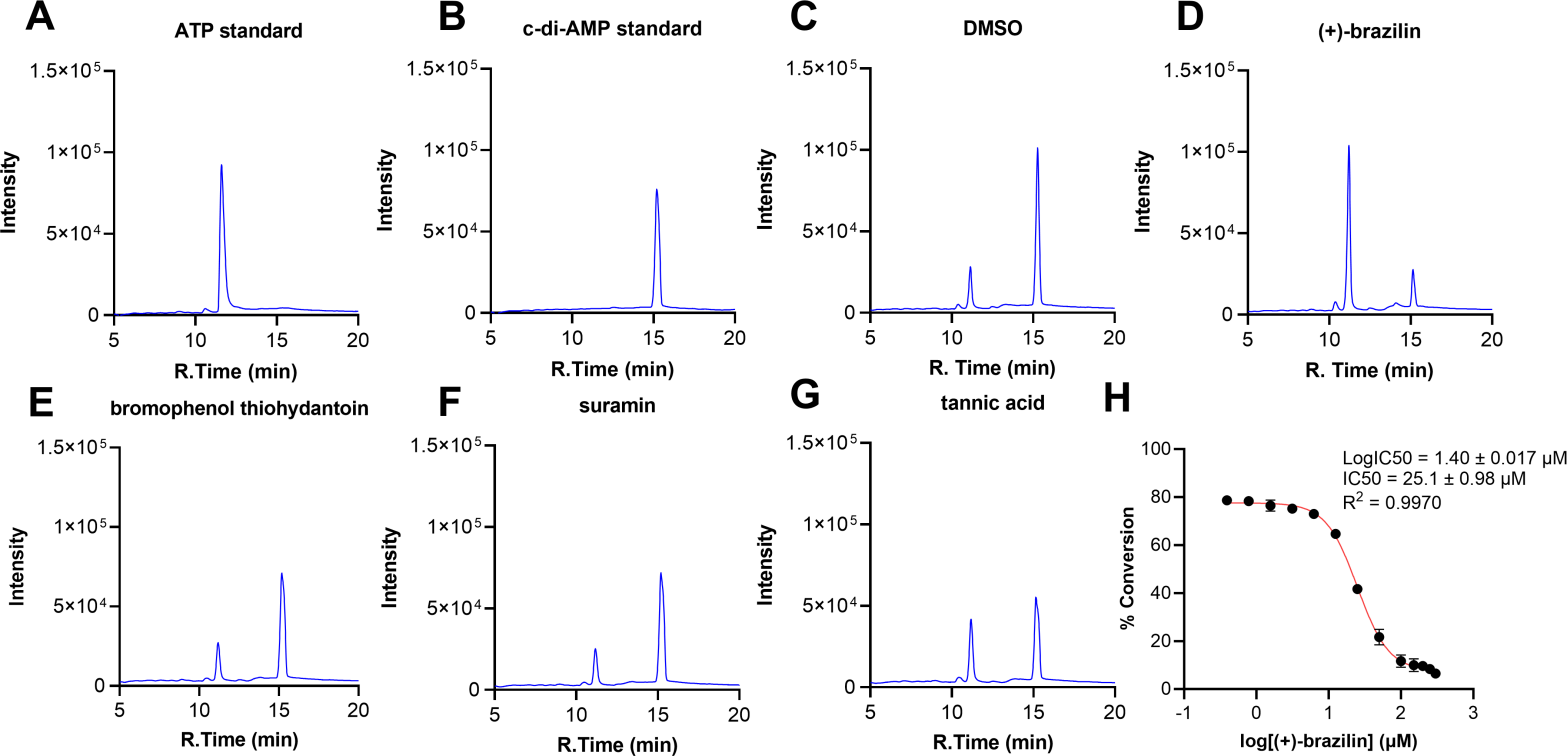
HPLC analysis to quantify the conversion of ATP into c-di-AMP by *sm*DAC. (**A and B**) Chromatograms of ATP (100 µM) and c-di-AMP (50 µM) standards, respectively. (**C and D**) Representative chromatograms of HPLC reactions treated without inhibitor (10% DMSO) or 50 µM (+)-brazilin, respectively. (**E–G**) Chromatograms of HPLC reactions treated with bromophenol thiohydantoin, suramin, and tannic acid at 50 µM, respectively. (**H**) IC_50_ curve generated from dose-dependent concentrations of (+)-brazilin against *sm*DAC. All HPLC experiments were repeated three times, and error bars denote the standard error of the mean. The raw data were exported from the LabSolutions Shimadzu software, analyzed, and plotted using GraphPad Prism 10.0.3.

Eleven “hits” were validated using HPLC to eliminate false positives from the coralyne assay. NSC numbers 8661, 123262, and 113490 inhibited c-di-AMP production by 87.1%, 79.6%, and 61.3%, respectively (Fig. S4). (+)-Brazilin (NSC 8661), showing the greatest inhibitory activity, was further studied in a dose-dependent manner. Representative chromatograms of ATP (100 µM) and c-di-AMP (50 µM) standards were used as references to validate retention times (Fig. 3A and B). Representative chromatograms of HPLC reactions treated with negative control and 50 µM (+)-brazilin again showed that (+)-brazilin significantly inhibited *sm*DAC’s enzymatic activity (Fig. 3C and D). Representative chromatograms of HPLC reactions treated with dose-dependent concentrations were also obtained (Fig. S5). Excitingly, (+)-brazilin inhibited the conversion of ATP into c-di-AMP in a dose-dependent manner with an IC_50_ value of 25.1 ± 0.98 µM (Fig. 3H; Table S1). These findings suggest that (+)-brazilin inhibits the conversion of ATP into c-di-AMP by *sm*DAC.

### Evaluation of known tmDisA inhibitors toward smDAC’s activity using HPLC

HPLC was also used to evaluate and compare bromophenol thiohydantoin, suramin, and tannic acid, all reported *tm*DisA inhibitors, to brazilin and 10% DMSO negative control. Representative chromatograms of HPLC reactions treated with 50 µM inhibitor or DMSO showed weak *sm*DAC inhibitory profiles for bromophenol thiohydantoin (3.7% inhibition), suramin (1.2% inhibition), and tannic acid (22.2% inhibition) compared to (+)-brazilin (77.8% inhibition) (Fig. 3E through G; Table S2). These findings suggest that bromophenol thiohydantoin, suramin, and tannic acid selectively target *tm*DisA over *sm*DAC.

### (+)-Brazilin is a non-competitive inhibitor of *sm*DAC determined by an HPLC steady-state Michaelis-Menten kinetics assay

**Fig 4:**
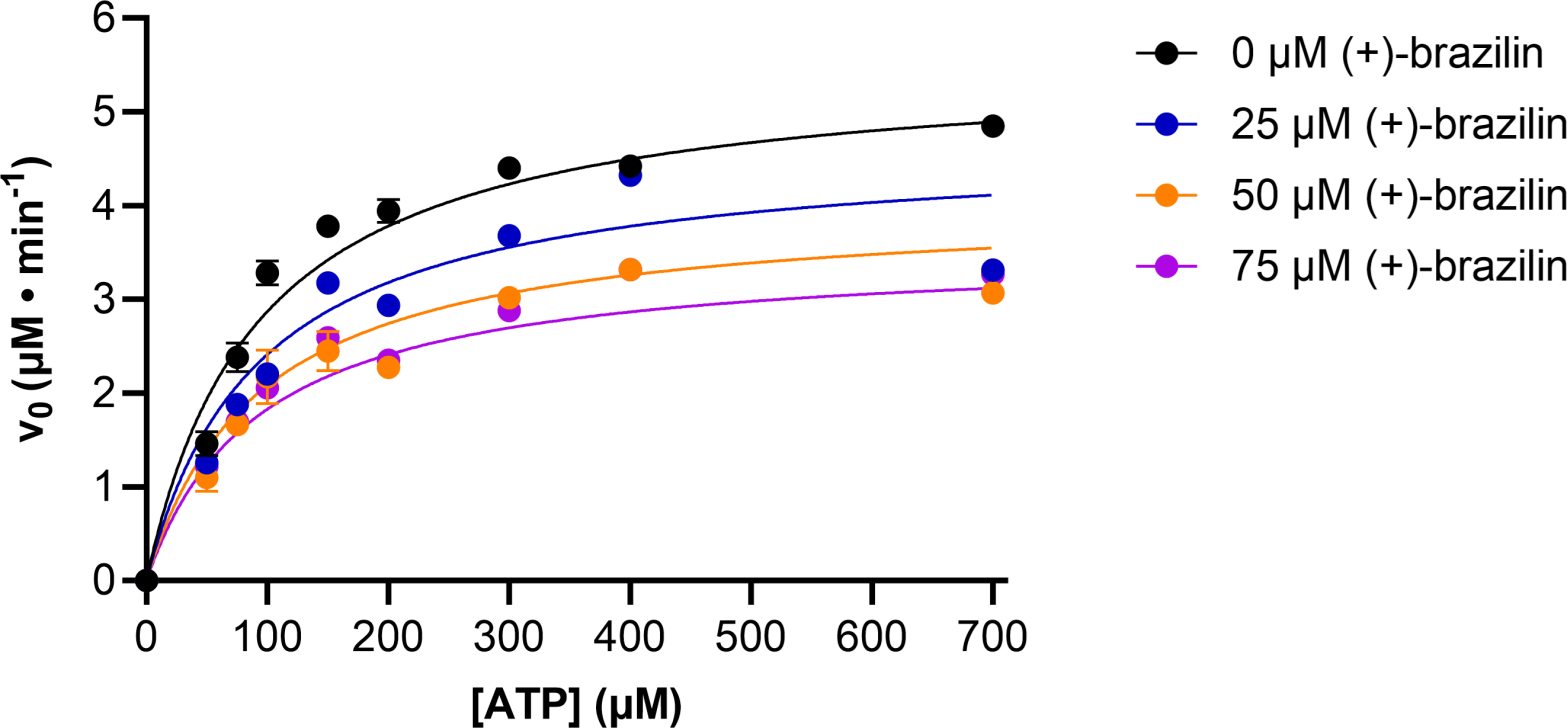
HPLC steady-state Michaelis-Menten kinetics of *sm*DAC’s enzymatic conversion of ATP to c-di-AMP in the presence of dose-dependent concentrations of (+)-brazilin (0, 25, 50, and 75 µM). Experiments were performed three times, and error bars represent the standard error of the mean. The data were fitted, plotted, and analyzed using a mixed model nonlinear regression curve in GraphPad Prism 10.0.3.

HPLC was employed to study the steady-state Michaelis-Menten kinetics of c-di-AMP synthesis without inhibitor and in the presence of 25, 50, and 75 µM (+)-brazilin. The maximum enzyme velocity of *sm*DAC’s activity (*V*_max_ = 5.54 ± 0.103 µM/min) and the Michaelis-Menten constant (*K*_*m*_ = 93.0 ± 3.98 µM), both in the absence of inhibitor, were obtained by plotting the initial velocities of *sm*DAC activity as a function of ATP concentration (Table 1). A decrease in the *V*_max_ with a constant *K*_*m*_ and an *α* value of 1.028 ± 0.2128 at dose-dependent concentrations of (+)-brazilin (25, 50, and 75 µM) suggest that (+)-brazilin is a non-competitive inhibitor and has comparable affinity to both the “free” *sm*DAC enzyme and the *sm*DAC-AMP complex (Fig. 4). The inhibition constant (*K*_*i*_ = 140.0 ± 27.13 µM) indicated that (+)-brazilin is not a potent inhibitor, yet still regulates *sm*DAC’s activity by targeting a site other than the substrate-binding site.

**TABLE 1:** Brazilin is a non-competitive inhibitor of *sm*DAC with *K*_*i*_ = 140.0 ± 27.13 µM^*a*^

Substrate	Product	*sm*DAC (µM)	*V*_max_ (µM/min)	*K*_*m*_ (µM)	*K*_*i*_ (µM)	*α*
ATP	c-di-AMP	10	5.54 ± 0.103	93.0 ± 3.98	140.0 ± 27.13	1.028 ± 0.2128

^*a*^ Kinetics parameters (*V*_max_, *K*_*m*_, *K*_*i*_, and *α*) were obtained from the mixed model nonlinear regression curve in GraphPad Prism 9.5.1.

### Binding profile of (+)-brazilin to *sm*DAC determined by intrinsic fluorescence

**Fig 5:**
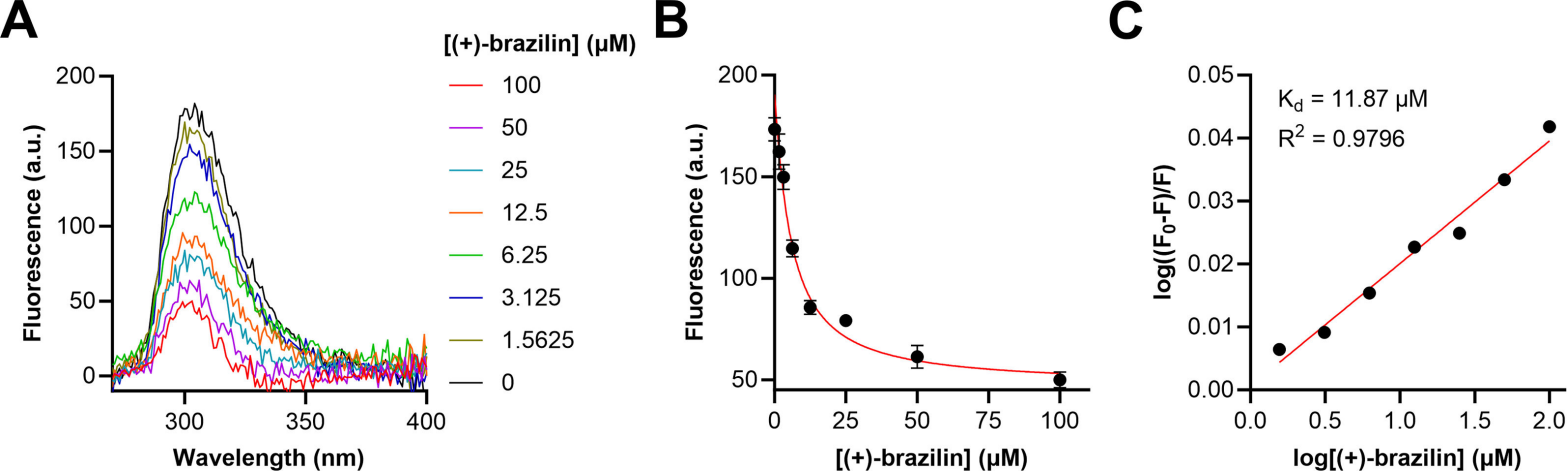
Intrinsic fluorescence binding assay of *sm*DAC. (**A**) Intrinsic fluorescence trace demonstrating the dose-dependent quenching effect of (+)-brazilin toward the tyrosine residue (*λ*_em_ = 303 nm), with *λ*_ex_ = 260 nm and *λ*_em_ = 270–450 nm. (**B**) Plot of fluorescence (a.u.) at 303 nm as a function of (+)-brazilin concentration. (**C**) Modified Sterne-Volmer plot with log $\frac{F_{0}-F}{F}$ as a function of log[µM (+)-brazilin]. *F*_0_ represents the fluorescence intensity without (+)-brazilin, and *F* represents the fluorescence intensity with (+)-brazilin. Three independent experiments were performed, and error bars represent the standard error of the mean. The data were analyzed and plotted using GraphPad Prism 10.0.3.

We employed an intrinsic fluorescence assay to gain insight into brazilin’s binding dissociation toward *sm*DAC. (+)-Brazilin quenched the intrinsic fluorescence of tyrosine (*λ*_em_ = 303 nm), one of the fluorescent residues in *sm*DAC, in a dose-dependent manner (Fig. 5A and B). From a Sterne-Volmer plot and using the modified Sterne-Volmer equation [Fig. 5C; equation (S1); Table S3], which used the fluorescence intensities corresponding to the varying concentrations of (+)-brazilin, the binding association constant *K*_*a*_ = 0.0842 µM and binding dissociation constant *K*_*d*_ = 11.87 µM were calculated [equation (S2)].

### (+)-Brazilin inhibits *S. mutans* UA159 single-species biofilm formation, glucan production, and eDNA levels

**Fig 6:**
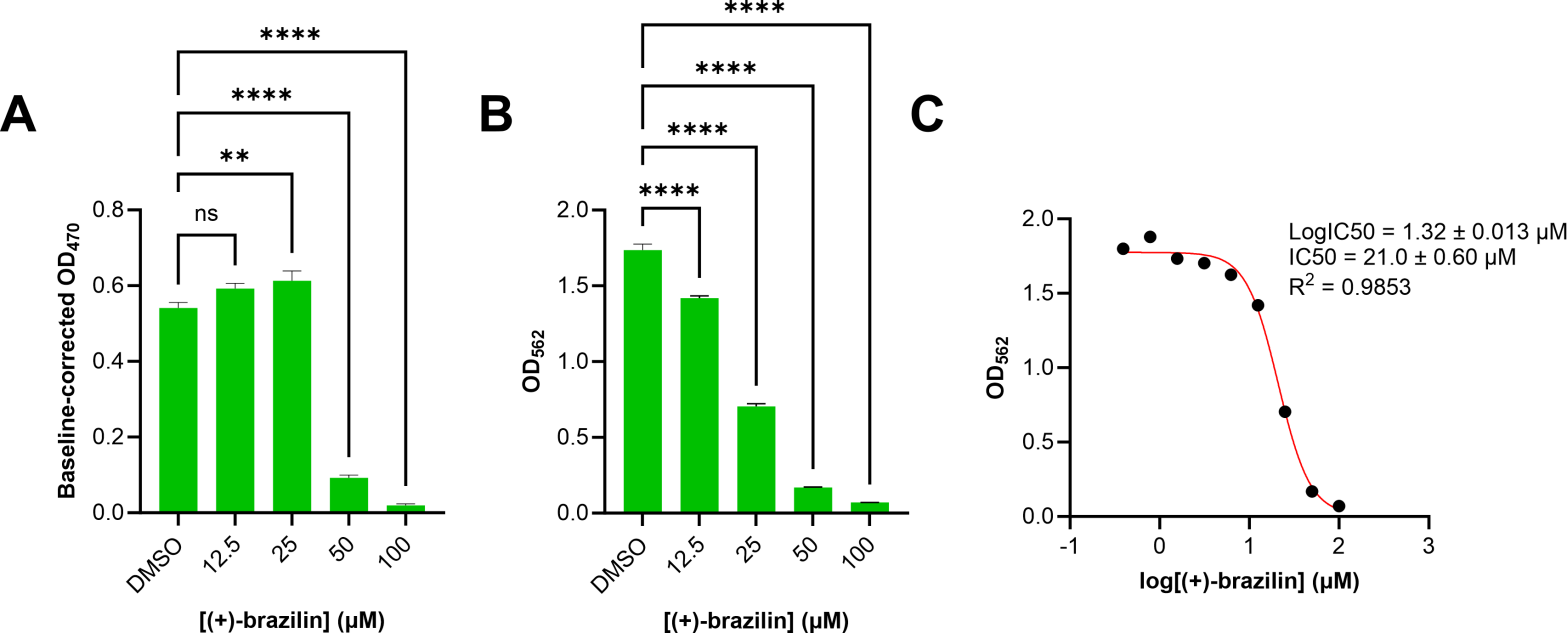
**(A and B**) Effect of dose-dependent concentrations of (+)-brazilin against the growth (OD_470_) and biofilm formation (OD_562_) of *S. mutans*, respectively. ****P value < 0.0001, **P value = 0.0058, and ns, not significant. Statistical analysis was performed by one-way ANOVA and Dunnett’s multiple comparisons test using GraphPad Prism 10.0.3. (**C**) (+)-Brazilin inhibits *S. mutans* biofilms with an IC_50_ = 21.0 ± 0.60 µM. The data were plotted and analyzed to obtain LogIC_50_ and IC_50_ values using GraphPad Prism 10.0.3. All experiments were performed three times on 96-well plates using 1% DMSO as a negative control, and error bars represent the standard error of the mean.

**Fig 7:**
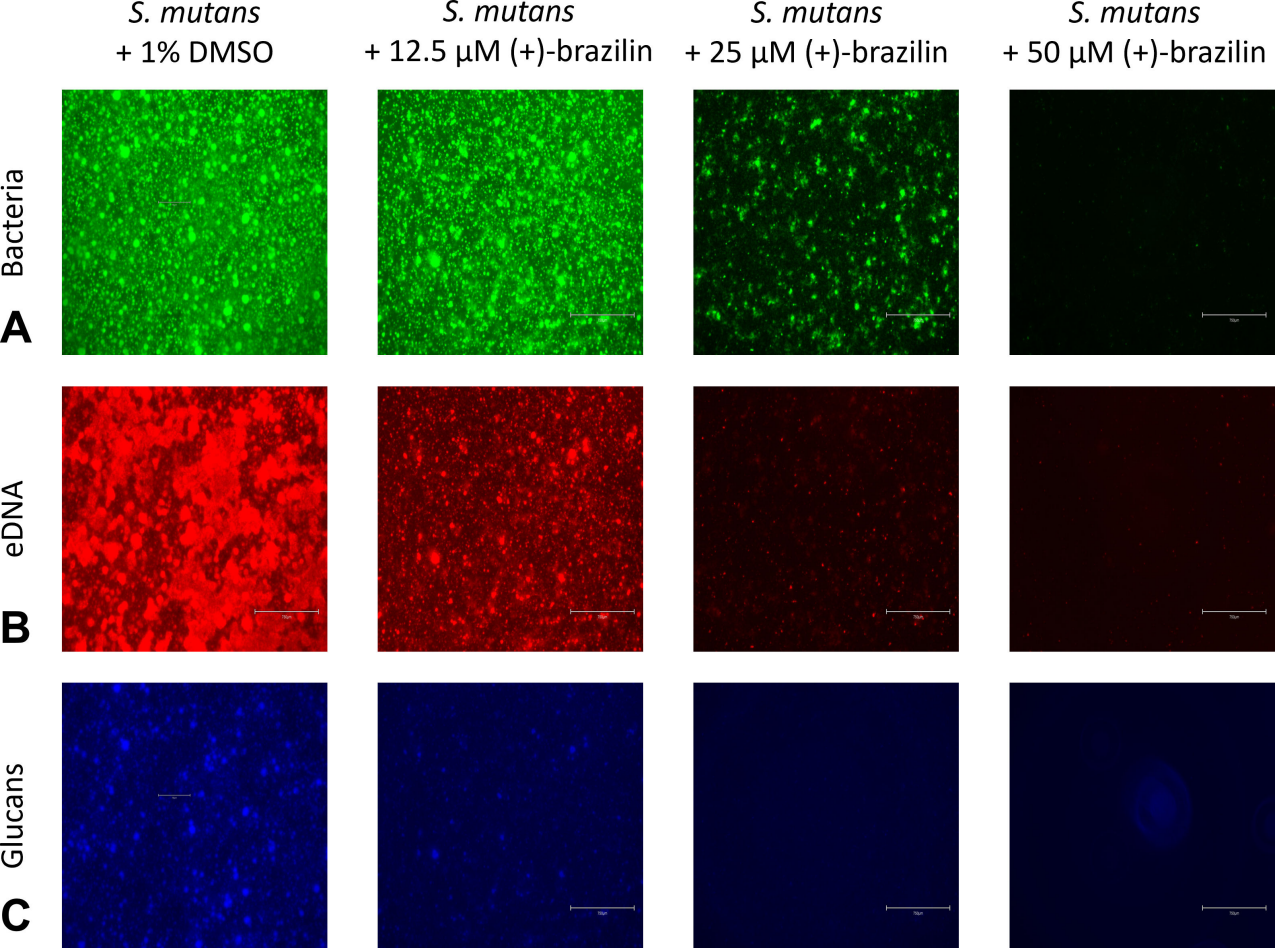
Representative fluorescence microscopy images of *S. mutans* biofilms after 16 h of treatment with dose-dependent concentrations of (+)-brazilin. (**A**) Bacterial cells (green) were stained with SYTO9 green fluorescent nucleic acid stain. (**B**) Extracellular DNA (red) was stained with propidium iodide. (**C**) Glucans (blue) were stained with dextran Cascade Blue conjugated dye. All experiments were performed three times on 96-well plates using 1% DMSO as a negative control. Scale bar = 750 µm.

**Fig 8:**
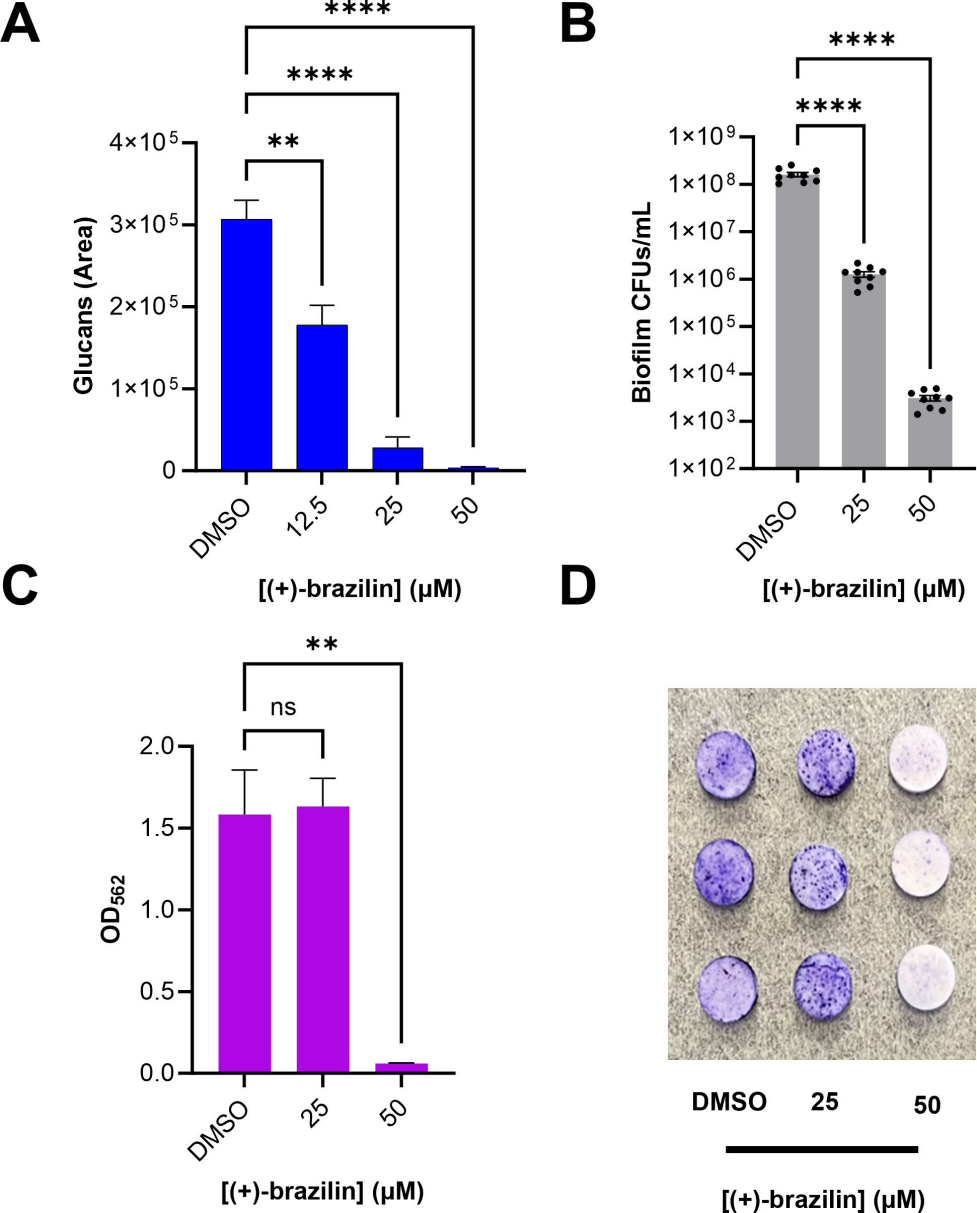
**(A**) Quantification of glucans obtained from fluorescence microscopy imaging using ImageJ 1.54j. ****P value < 0.0001 and **P value = 0.0024. Statistical analysis was performed by one-way ANOVA and Dunnett’s multiple comparisons test using GraphPad Prism 10.0.3. (**B**) Enumeration of colony-forming units in Brain Heart Infusion agar from *S. mutans* biofilms when treated with (+)-brazilin (25 and 50 µM) compared to 1% DMSO. ****P value < 0.0001. Statistical analysis was performed by one-way ANOVA and Dunnett’s multiple comparisons test using GraphPad Prism 10.0.3. (**C and D**) Crystal violet biomass quantification and images of *S. mutans* biofilms formed on vertically suspended HA discs when treated with (+)-brazilin (25 and 50 µM) compared to 1% DMSO. **P value = 0.0021 and ns, not significant. Statistical analysis was performed by one-way ANOVA and Dunnett’s multiple comparisons test using GraphPad Prism 10.0.3. All experiments were performed three times on 96-well plates using 1% DMSO as a negative control, and error bars represent the standard error of the mean.

We also evaluated (+)-brazilin’s anti-biofilm activity against *S. mutans*. (+)-Brazilin inhibited biofilm formation of *S. mutans* with an IC_50_ value of 21.0 ± 0.60 µM, determined by crystal violet staining (Fig. 6B and C). Fluorescence imaging with SYTO9 to stain bacterial cells showed a drastic reduction in *S. mutans* biofilms treated with 25 µM (+)-brazilin and near complete inhibition with 50 µM (+)-brazilin when compared to 1% DMSO negative control (Fig. 7, panel A). Fluorescence imaging with propidium iodide to visualize eDNA showed a significant reduction in eDNA levels with 12.5 µM (+)-brazilin and nearly complete absence of eDNA with 25 µM (+)-brazilin when compared to 1% DMSO negative control (Fig. 7, panel B). The presence of glucans, which were tracked with dextran conjugated Cascade Blue dye, was significantly reduced (40%–50%) with 12.5 µM (+)-brazilin, and almost complete reduction (>90%) was observed with 25 µM (+)-brazilin when compared to 1% DMSO negative control (Fig. 7, panel C and Fig. 8A). These findings suggest that (+)-brazilin is an *S. mutans* biofilm inhibitor that prevents the synthesis of glucans and reduces eDNA levels, two crucial attributes for *S. mutans* to form robust biofilm matrices. To further evaluate the effect of (+)-brazilin on *S. mutans*, we enumerated bacterial colony-forming units (CFUs) from biofilms.

CFUs of *S. mutans* biofilms in the order of ~10^8^ CFUs/mL can be recovered from the untreated control. Treatment with 25 µM (+)-brazilin inhibited bacterial viability by ~10^2^ , whereas treatment with 50 µM (+)-brazilin inhibited by ~10^5^ (Fig. 8B). These findings support that (+)-brazilin inhibits *S. mutans* biofilm formation *in vitro*, as observed by the reduction of biofilm biomass from the crystal violet assay and the reduction of CFUs.

### (+)-Brazilin inhibits *S. mutans* UA159 colonization on hydroxyapatite discs

To glean if (+)-brazilin can inhibit biofilms on lateral surfaces, we used an HA disc assay as a representative model of the tooth surface. Treatment with 25 µM (+)-brazilin did not statistically inhibit biofilm formation on the HA discs compared to the negative control discs; however, treatment with 50 µM (+)-brazilin inhibited biofilm formation by >90% (Fig. 8C). Photographs of the HA discs stained in crystal violet and taken prior to dissolution with 30% acetic acid align with the quantitative results (Fig. 8D).

### (+)-Brazilin does not affect the growth of oral streptococcal species at low micromolar concentrations *in vitro*

**Fig 9:**
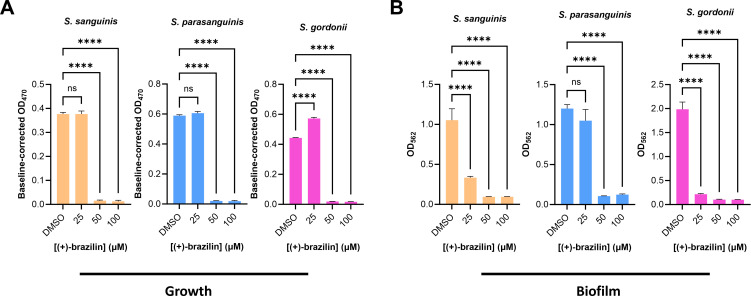
**(A and B**) Effect of dose-dependent concentrations of (+)-brazilin against the growth (OD_470_) and biofilm formation (OD_562_) of oral commensal streptococci, respectively. ****P value < 0.0001 and ns, not significant. Statistical analysis was performed by one-way ANOVA and Dunnett’s multiple comparisons test using GraphPad Prism 10.0.3. All experiments were performed three times on 96-well plates using 1% DMSO as a negative control, and error bars represent the standard error of the mean.

To determine if (+)-brazilin exhibits selective inhibition toward oral *streptococcal* biofilms over bacterial growth, we evaluated (+)-brazilin in a dose-dependent manner, *in vitro*. No decrease in cell viability of *S. mutans* was detected with 12.5 and 25 µM (+)-brazilin; however, there was selective biofilm inhibition of 18.2% and 59.4%, respectively, at these concentrations. There was a significant decrease in cell viability with 50 µM (84.9% inhibition) and 100 µM (96.8% inhibition) (+)-brazilin when compared to 1% DMSO negative control (Fig. 6A). Three commensal streptococcal strains (*Streptococcus sanguinis*, *Streptococcus parasanguinis*, and *Streptococcus gordonii*) were treated with 25, 50, and 100 µM (+)-brazilin to determine its effect on cell viability and biofilms, *in vitro*. No significant difference in cell viabilities of *S. sanguinis* and *S. parasanguinis* was observed when treated with 25 µM (+)-brazilin; however, an increase was observed for *S. gordonii* at the same concentration compared to 1% DMSO negative control (Fig. 9A). A near complete reduction in cell viability was observed for *S. sanguinis*, *S. parasanguinis*, and *S. gordonii* at 50 µM (+)-brazilin (Fig. 9A). Interestingly, biofilms of *S. sanguinis* (68.4% inhibition) and *S. gordonii* (89.1% inhibition) were significantly inhibited at 25 µM (+)-brazilin, yet for *S. parasanguinis* (91.2% inhibition), this effect was observed at 50 µM (+)-brazilin (Fig. 9B). In summary, (+)-brazilin at low micromolar concentrations did not reduce the growth of all three commensal streptococci but inhibited their biofilm formation above 50 µM. (+)-Brazilin selectively inhibited *S. mutans* biofilm formation while preserving the growth at 25 µM.

## DISCUSSION

The focus of this study was to discover a small molecule inhibitor of *sm*DAC to inhibit the conversion of ATP to c-di-AMP, a secondary messenger that contributes to biofilm formation in *S. mutans* (12). To identify potential inhibitors, we first established biochemical assays to observe *sm*DAC’s catalytic activity.

It is still a mystery that DAC enzymes are selective toward metal cofactors. *sm*DAC falls into the family of CdaA enzymes, which are known to be enzymatically active in the presence of Co^2+^ or Mn^2+^. We determined that *sm*DAC has optimal enzymatic activity in the presence of 5–10 mM Mn^2+^, which compares to what has been reported for CdaA of *Staphylococcus aureus* (50). Interestingly, *sm*DAC converted ATP into c-di-AMP in the presence of 10 mM Co^2+^, but the turnover observed was significantly less than with Mn^2+^. The larger ionic radius of Mn^2+^ (91 pm) compared to Co^2+^ (70 pm) may better stabilize the metal cation inside *sm*DAC’s catalytic site. It is also likely that the Ca^2+^ (100 pm) ion is too large for the coordination required at *sm*DAC’s active site. However, comparing ionic radii alone does not explain this phenomenon since Mg^2+^ (79 pm) has an ionic radius between that of Co^2+^ and Mn^2+^. Based on our HPLC assay, *sm*DAC showed significantly higher selectivity toward Mn^2+^ over Co^2+^, and additional studies are needed to better understand the selective coordination chemistry of Mn^2+^ versus Co^2+^ in the CdaA enzyme family.

We used the coralyne assay to conduct an *in vitro* high-throughput screening of an NCI natural product library to obtain “hits” that exhibited *sm*DAC inhibitory activity. One of the compounds with the potential to inhibit *sm*DAC was (+)-brazilin, a tetracyclic homoisoflavanoid isolated from the heartwood of *C. sappan*. Since (+)-brazilin, along with many other compounds, strongly competed with potassium iodide and quenched the fluorescence of the fluorophore coralyne, an IC_50_ curve from the coralyne assay would not accurately represent a dose-dependent inhibition of *sm*DAC’s enzymatic activity. (+)-Brazilin contains phenolic groups and aromatic groups, which are capable of quenching fluorophores (51). Therefore, a quantitative HPLC assay was pursued to confirm that (+)-brazilin can inhibit *sm*DAC’s function.

(+)-Brazilin inhibited c-di-AMP formation in a dose-dependent manner, with an enzymatic inhibitory IC_50_ = 25.1 ± 0.98 µM. Interestingly, 50 µM bromophenol thiohydantoin and 50 µM suramin, two reported *tm*DisA inhibitors, did not show significant inhibitory activity toward *sm*DAC, with inhibitory profiles of 3.7% and 1.2%, respectively. However, 50 µM tannic acid, another reported *tm*DisA inhibitor, did show some inhibitory activity toward *sm*DAC, with an inhibitory profile of 22.2%. (+)-Brazilin at the same concentration exhibited an *sm*DAC inhibitory profile of 77.8%. (+)-Brazilin possesses three phenol groups and a chiral tertiary alcohol, is low molecular weight (MW = 286.28 Da), and is more drug-like than tannic acid (MW = 1,701.19 Da). Although the catalytic domain is conserved among all DAC enzymes, the observation that the reported *tm*DisA inhibitors are not potent inhibitors of *sm*DAC revealed that DisA and CdaA enzymes can be targeted selectively. To further support that (+)-brazilin’s inhibitory effect in c-di-AMP production can be observed not only via a biochemical assay but also directly from *S. mutans*, we plan in subsequent studies to optimize our *in vitro* biofilm assay to quantify intracellular c-di-AMP levels with and without treatment of (+)-brazilin. Studies have evaluated c-di-AMP levels from a planktonic overnight culture of *S. mutans* lacking sucrose (12, 52); however, it would be interesting to measure intracellular c-di-AMP levels in biofilm-promoting conditions, particularly with sucrose, as described in our work.

Furthermore, we obtained preliminary evidence of binding to *sm*DAC by (+)-brazilin quenching tyrosine’s intrinsic fluorescence in a dose-dependent manner. Steady-state Michaelis-Menten kinetics suggest that (+)-brazilin may inhibit *S. mutans* biofilms by non-competitive inhibition of *sm*DAC activity, although (+)-brazilin has a weak affinity to *sm*DAC, with a *K*_*i*_ =140.0 ± 27.13 µM. Our future work aims to identify the binding site of (+)-brazilin on *sm*DAC. Since we have learned that (+)-brazilin is a non-competitive inhibitor, (+)-brazilin is likely interacting with tyrosine residues outside of the AMP-binding pocket. Binding studies of DAC enzymes are scarce in the literature, and we hope to see more development soon. Although (+)-brazilin’s binding profile is still not well understood, we employed an established intrinsic fluorescence assay to gain preliminary insight and plan to develop a more robust secondary binding assay to better characterize the binding kinetics of *sm*DAC inhibitors. Co-crystallization of (+)-brazilin with *sm*DAC would identify which structural motifs are important for binding, and further *in silico* structure-activity-relationship studies may lead to the discovery of potent (+)-brazilin-like compounds more appropriate for downstream stages of the drug development pipeline.

(+)-Brazilin was also evaluated for its bactericidal and anti-biofilm properties toward pathogenic *S. mutans* and oral commensal streptococcal species. Although (+)-brazilin showed bactericidal effects in all streptococci when examined above 50 µM, there was considerable selectivity for biofilm inhibition of *S. mutans* at or below 25 µM without affecting its planktonic growth. Interestingly, 25 µM (+)-brazilin increased the growth of *S. gordonii* while inhibiting its biofilm, suggesting that (+)-brazilin may be favoring a pathway in *S. gordonii* for the survival of planktonic cells over its ability to develop biofilms. A similar effect was observed for *S. sanguinis* at 25 µM (+)-brazilin. At the same concentration, (+)-brazilin did not show a significant difference in the survival of *S. parasanguinis* planktonic cells or biofilm formation compared to the negative control. It is likely that (+)-brazilin’s phenol groups may be contributing to its bactericidal activity and low selectivity across oral microbes (53, 54). Since we have strong evidence that (+)-brazilin targets *sm*DAC, a key player in *S. mutans* biofilm formation, we are optimistic that (+)-brazilin’s selectivity toward inhibiting *S. mutans* biofilms and potency can be improved by generating and testing a library of analogs and derivatives with modifications to the phenol groups.

Fluorescence microscopy assays of *S. mutans* biofilm treated with (+)-brazilin showed a drastic reduction of eDNA and glucans, both essential components in the oral biofilm matrix. Although the mechanisms of eDNA production and regulation are still being unraveled, eDNA has been shown to participate in the initial attachment of oral microorganisms and further stabilize the biofilm matrix (55). It is unknown how c-di-AMP regulates eDNA production in *S. mutans*. In *S. aureus*, high levels of c-di-AMP have been shown to promote autolysis, eDNA levels, cell wall integrity, and biofilm formation (56–58). It is plausible that c-di-AMP regulates autolysis and eDNA release in *S. mutans* using a similar pathway. (+)-brazilin’s regulatory role in eDNA production is unclear and further studies are needed to understand why a decrease in c-di-AMP production leads to diminishing eDNA release. Furthermore, (+)-brazilin’s regulatory role in glucan production is likely attributed to inhibiting *sm*DAC, an enzyme upstream that has been shown to regulate the expression of *gtfB*.

(+)-Brazilin significantly inhibited *S. mutans* colonization (CFUs/mL) from pre-formed biofilms, with the greatest reduction of CFUs when treated with 50 µM (+)-brazilin. This result aligned with the decrease in biofilms in 96-well polystyrene plates. However, this crystal violet staining assay does not consider biofilms formed on a rough 3-D surface, such as those on the buccal, lingual, and proximal surfaces of teeth. We therefore employed an HA disc assay and observed a significant inhibition of biofilms formed on the lateral surfaces of HA discs at the same concentration. It was not surprising to see no reduction in biofilms on HA discs treated with 25 µM (+)-brazilin since *S. mutans*, along with other bacterial species, are able to genetically adapt, colonize, adhere, and construct biofilms more readily on rough, porous surfaces, such as an HA disc, rather than on smoother surfaces with less surface area, such as a polystyrene plate (59–61). Future studies will be employed to glean how (+)-brazilin regulates biofilm formation on various surfaces and if (+)-brazilin may be affecting the mechanisms of adhesion at varying concentrations. This can be accomplished by incorporating a mixed-species biofilm model on human saliva-coated HA discs, by co-infecting rats with *S. mutans* and commensal streptococci, and by enumerating CFUs of each bacterial species, thus providing a more representative model of the oral microbiome.

(+)-Brazilin is relatively expensive and impractical to isolate from *C. sappan* for further examination. However, its intriguing scaffold has recently generated much attention in pharmacological research (30, 32, 34, 39, 40, 42, 62–64). Although (+)-brazilin can be synthesized using various methods (65–73), many reported syntheses involve several steps with low yields. We are currently improving the synthetic accessibility of (+)-brazilin to provide a more efficient route of access to (+)-brazilin and similar compounds.

In summary, we have characterized a first-generation inhibitor of *sm*DAC. (+)-Brazilin is a known natural product rich in biological applications. Our studies demonstrated a low-micromolar selective anti-biofilm effect toward *S. mutans* by targeting and non-competitively inhibiting the function of *sm*DAC. We acknowledge that (+)-brazilin presents as a broad-spectrum inhibitor of oral streptococci; however, we are very optimistic that the selectivity toward *S. mutans* biofilms can be increased through structural modifications. There is also a growing interest in non-competitive inhibitors for the drug discovery of ATP-binding proteins, especially since ATP concentrations are generally high in metabolically active cellular compartments (74, 75). The present study examined (+)-brazilin’s regulatory role on *sm*DAC, but more importantly, it provided a robust toolbox for the discovery and characterization of potent inhibitors of *sm*DAC. We hope this novel launching pad will advance the development of therapeutics for the prevention and treatment of dental caries. Similar to the elucidation of other potent anti-biofilm therapeutics developed within our lab, we plan to encapsulate a more potent and selective analog of (+)-brazilin into a polymeric material to study its drug-release kinetics and anti-cariogenic activity in an *in vivo* rat caries model in order to explore its full therapeutic potential (76).

## MATERIALS AND METHODS

### *sm*DAC expression and purification

A gene coding for recombinant *sm*DAC (from 116aa to 265aa) was amplified with primer set DAC-F (GATCAGCTAGCCTGAGTGATGATGAAAAATTAGTTGCC) and DAC-R (TCACTCGAGTTTAATGAAATGCTCCCGCAATAA)(Restriction enzyme sites are underlined). The PCR products were digested by restriction enzymes Nhe1 and Xho1 and then inserted into vector pET-21a for protein expression in *Escherichia coli* BL21 (DE3). The colonies were selected by ampicillin (100 µg/mL) and confirmed by PCR and DNA sequencing.

The overnight culture of *E. coli* BL21 (DE3) containing pET21-*sm*DAC plasmid was transferred into 1 L of freshly prepared Luria-Bertani (LB) medium at a ratio of 1:100 in a 2 L shake flask and grown at 37°C and 250 rpm until the OD_600_ reached 0.6. Then, 0.1 mM isopropyl-β-D-thiogalactopyranoside was added to induce the protein at 18°C, 250 rpm overnight.

The induced cells were harvested after centrifuging at 5,000 rpm for 10 min and then suspended in 30 mL lysis buffer (20 mM Tris, pH 8.0, 500 mM NaCl, and 30 mM imidazole). After sonicating the cells for 6 min (5 s on pulse and 10 s on pause) to release the protein, the cells were centrifuged at 16,000 rpm for 30 min at 4°C to obtain a clear lysate. The protein was purified through HisTrap high performance column (GE) by AKTA prime system (GE) with elution buffer (20 mM Tris, pH 8.0, 500 mM NaCl, and 500 mM imidazole). The eluted protein was further isolated in 1× PBS buffer using size-exclusive chromatographic column Superdex 200 10/300 GL (GE). Thereafter, the purified protein was loaded on an SDS-PAGE to check the purity, and Precision Plus Protein Dual Color standards (Bio-Rad) were used to indicate the protein size.

### Bacterial strains and culture conditions

Streptococcus strains, including *S. mutans* UA159, *S. sanguinis* SK36, *S. gordonii* DL1, and *S. parasanguinis* FW213, were grown statically for 24 h at 37°C and 5% CO_2_ in freshly autoclaved Todd Hewitt Broth (THB) (5 mL).

### Single-species biofilm inhibition assay

*S. mutans* UA159, *S. sanguinis* SK36, *S. gordonii* DL1, and *S. parasanguinis* FW213 were independently grown for 24 h at 37°C and 5% CO_2_ in freshly autoclaved THB (5 mL). The overnight culture was then diluted 1:100 into Brain Heart Infusion (BHI) medium (1 mL) containing 1% sucrose and 1% DMSO or inhibitor in 96-well plates. After incubation for 16 h at 37°C and 5% CO_2_, the planktonic cells were quantified at OD_470_, and the biofilms were gently washed with H_2_O three times, dried, and then stained with 0.1% crystal violet (200 µL) for 30 min. The plates were then gently washed with H_2_O three times, treated with 30% acetic acid (200 µL), and placed on an orbital shaker (400 rpm) at ambient temperature for 1 h. *S. mutans* biofilms were then further diluted 1:1 with 30% acetic acid before quantifying the biomass at OD_562_, while commensal strains were quantified without further dilution. Three independent experiments were performed in quadruplicate, and error bars represent the standard error of the mean (SEM). Baseline subtraction from wells without bacterial cultures was performed to calculate the growth at OD_470_ for all experiments. Optical densities were measured using an Agilent BioTek 800TS Absorbance Reader equipped with 470 and 562 nm filters.

### Colony-forming units assay

*S. mutans* UA159 was grown for 24 h at 37°C and 5% CO_2_ in freshly autoclaved THB (5 mL). The overnight culture was then diluted 1:100 into BHI medium (1 mL) containing 1% sucrose and 1% DMSO or inhibitor in 96-well plates. After incubation for 16 h at 37°C and 5% CO_2_, the biofilms were gently washed with H_2_O three times, dried, and then treated with 1× PBS (100 µL). Dispersed biofilms (100 µL) were serially diluted in 1× PBS, plated in triplicate on BHI agar plates (100 × 15 mm) using sterilized EZ-Spread Plating Beads (Biomyx Technology, Inc.), and incubated for 24 h at 37°C and 5% CO_2_. The total number of viable bacterial cells (CFUs) was then determined. Three independent experiments were performed, and error bars represent the SEM.

### Hydroxyapatite disc biofilm inhibition assay

*S. mutans* UA159 was grown for 24 h at 37°C and 5% CO_2_ in freshly autoclaved THB (5 mL). Orthodontic metal pliers were sterilized in absolute ethanol, flamed, and then used to vertically insert hydroxyapatite (HA) discs (Dense Ceramic Hydroxyapatite Disc, 5 mm diameter × 2 mm thickness, Clarkson Chromatograph Products, Inc., South Williamsport, PA, USA) into 96-well plates. The overnight culture was then diluted 1:100 into BHI medium (1 mL) containing 1% sucrose and 1% DMSO or inhibitor; biofilm medium (300 µL) was carefully added into each well. After incubation for 24 h at 37°C and 5% CO_2_, the biofilm medium from each well was pipetted out, and biofilm medium (300 µL) [from a fresh overnight culture diluted 1:100 into BHI medium (1 mL) containing 1% sucrose and 1% DMSO or inhibitor] was carefully added into each well. After incubation for 20 h at 37°C and 5% CO_2_, the biofilm medium from each well was pipetted out, and the biofilm-coated HA discs were carefully removed using sterilized orthodontic pliers and gently dipped into individual centrifuge tubes (50 mL) containing milli-Q water. The HA discs were then vertically inserted into new wells and stained with 0.1% crystal violet (300 µL) for 30 min. The crystal violet was then pipetted out, and milli-Q water (300 µL) was carefully added to the wells (repeated 10 times). The HA discs were then carefully placed vertically onto Kimwipes to dry for 15 min and then placed flat for a picture to be taken. The HA discs were then vertically inserted into new wells, treated with 30% acetic acid (250 µL), and placed on an orbital shaker (250 rpm) at ambient temperature for 1 h. Dissolved biofilms (200 µL) were pipetted into new wells and then further diluted 1:1 with 30% acetic acid (100 µL; final volume = 200 µL) into new wells. Another 1:1 dilution with 30% acetic acid (100 µL; final volume = 200 µL) was done into new wells, which were then used to quantify the biomass at OD_562_. Three independent experiments were performed, and error bars represent the SEM. Optical densities were measured using an Agilent BioTek 800TS Absorbance Reader equipped with a 562 nm filter.

### Fluorescence microscopy of *S. mutans* biofilms

*S. mutans* UA159 was grown for 24 h at 37°C and 5% CO_2_ in freshly autoclaved THB (5 mL). The overnight culture was then diluted 1:100 into BHI medium (1 mL) containing 1% sucrose, 1% DMSO or inhibitor, and 1 µM dextran Cascade Blue (10,000 MW, anionic, lysine flexible) in 96-well plates. After incubation for 16 h at 37°C and 5% CO_2_, the biofilms were gently washed with H_2_O three times, dried, and wells were stained with 1× PBS (200 µL) containing 1 µM SYTO9 green fluorescent nucleic acid stain and 2 µg/mL propidium iodide. After gentle shaking for 10 min, while covered in aluminum foil, the stained biofilms were visualized with a 4× objective using an EVOS M5000 Imaging System. GFP green light, RFP red light, and DAPI blue light were used to visualize bacterial cells, eDNA, and glucans, respectively. Fluorescence microscopy images of glucans were quantified using ImageJ 1.54j. Images are representative of three independent experiments performed on different days.

### Metal cofactor determination for *sm*DAC activity using HPLC

The HPLC assay was optimized from a reported procedure, as described (22). To determine which cofactor (Mn^2+^, Mg^2+^, Ca^2+^, or Co^2+^) is optimal for *sm*DAC’s catalytic activity, reactions of 400 µL containing 100 µM ATP, 5 µM *sm*DAC, and 10% DMSO in reaction buffer (40 mM HEPES, pH 7.5, 100 mM NaCl, and 10 mM metal chloride) were incubated statically at 30°C for 4 h on a heat block. The reactions were then heat-shocked at 95°C for 6 min, let stand at room temperature for 15 min, transferred to 3 KD centrifuge spin filters (VWR, Cat# 82031-344), and centrifuged at 10,000 rpm for 30 min, and the filtrates (300 µL) were transferred to HPLC vials. The filtrates were injected (10 µL) and ATP and c-di-AMP peaks were detected using a Kinetex 5 µm C18 100 Å, LC Column 150 × 4.6 mm (Phenomenex, Cat# 00F-4601-E0). ATP and c-di-AMP signals were compared to commercial standards. The HPLC method is as follows: 0–22 min (1%–13% acetonitrile/100 mM TEAA), 22–24.5 min (13%–90% acetonitrile/100 mM TEAA); 24.5–28.5 min (90% acetonitrile/100 mM TEAA); 28.5–28.51 min (90%–1% acetonitrile/100 mM TEAA); 28.51–36 min (1% acetonitrile/100 mM TEAA). Flow rate: 0.5 mL/min. Oven temperature: 30°C. Signals were analyzed using a 254 nm UV detector.

### Screening of natural product libraries using the coralyne assay

The coralyne assay was optimized from a reported procedure, as described (22,77). Briefly, natural product stock solutions (5 or 10 mM) in DMSO (20 polyphenol compounds from NCI) were screened at 100 µM for *sm*DAC inhibition. Reactions of 300 µL containing 10 µM coralyne chloride, 10 mM KI, 100 µM ATP, 10% DMSO or inhibitor, and 5 µM *sm*DAC in reaction buffer (40 mM HEPES, pH 7.5, 100 mM NaCl, and 10 mM MnCl_2_) were set up at 20°C in 96-well plates. Once (+)-brazilin was identified as a “lead,” the coralyne assays were performed at 30°C on a heat block before being transferred to a 96-well plate. A Cary Eclipse Fluorescence Spectrophotometer equipped with a microplate reader accessory was used to measure the fluorescence intensity with excitation and emission wavelengths of 420 and 475 nm, respectively, for 4 h with 0.30-min intervals. Three independent experiments were performed, and error bars represent the standard error of the mean.

### HPLC enzymatic inhibition assay

The HPLC assay was optimized from a reported procedure, as described (22), using compound stock solutions in DMSO. Reactions of 400 µL containing 100 µM ATP, 5 µM *sm*DAC, and 10% DMSO in reaction buffer (40 mM HEPES, pH 7.5, 100 mM NaCl, and 10 mM MnCl_2_) were incubated statically at 30°C for 4 h on a heat block. The reactions were then heat-shocked at 95°C for 6 min, let stand at room temperature for 15 min, transferred to 3 KD centrifuge spin filters (VWR, Cat# 82031-344), and centrifuged at 10,000 rpm for 30 min, and the filtrates (300 µL) were transferred to HPLC vials. The filtrates were injected (10 µL) and ATP and c-di-AMP peaks were detected using a Kinetex 5 µm C18 100 Å, LC Column 150 × 4.6 mm (Phenomenex, Cat# 00F-4601-E0). Percent c-di-AMP produced from each reaction was determined based on the ratio of the peak area of c-di-AMP to the peak area of ATP. ATP and c-di-AMP signals were compared to commercial standards. The HPLC method is as follows: 0–22 min (1%–13% acetonitrile/100 mM TEAA), 22–24.5 min (13%–90% acetonitrile/100 mM TEAA); 24.5–28.5 min (90% acetonitrile/100 mM TEAA); 28.5–28.51 min (90%–1% acetonitrile/100 mM TEAA); and 28.51–36 min (1% acetonitrile/100 mM TEAA). Flow rate: 0.5 mL/min. Oven temperature: 30°C. Signals were analyzed using a 254 nm UV detector.

### HPLC steady-state Michaelis-Menten kinetics assay

The steady-state Michaelis-Menten kinetics of *sm*DAC activity was studied with and without the presence of (+)-brazilin. Individual discontinuous first-order time course enzymatic reactions containing 10 µM *sm*DAC and 10% DMSO or inhibitor [25, 50, or 75 µM (+)-brazilin] were performed at 50, 75, 100, 150, 200, 300, 400, and 700 µM ATP. The data were fitted using a mixed-model inhibition mode on GraphPad Prism, which uses a general equation to consider competitive, non-competitive, and uncompetitive types of inhibition.

#### Example of enzymatic reaction for 50 µM ATP without inhibitor

To a 1.5 mL centrifuge tube (Tube A), 80 µL of 5× reaction buffer (200 mM HEPES, pH 7.5, 500 mM NaCl, and 50 mM MnCl_2_), 40 µL DMSO, 220 µL milli-Q H_2_O, and 20 µL 1 mM ATP were added. The tube was vortexed for 15 s, centrifuged at 3,000 rpm for 30 s, and then incubated statically on a heat block at 30°C for 30 min. To another 1.5 mL centrifuge tube (Tube B), 40 µL of 100 µM *sm*DAC was added, which was incubated on the same heat block at 30°C for 30 min, simultaneously with Tube A. Four 1.5 mL centrifuge tubes (Tubes C–F), each containing 380 µL of 1× reaction buffer (40 mM HEPES, pH 7.5, 100 mM NaCl, and 10 mM MnCl_2_), were incubated statically on a separate heat block at 95°C for 30 min, simultaneously with Tubes A and B. The reaction was initiated by pipetting the contents from Tube A into Tube B and allowing the reaction (400 µL) to progress at 30°C. The reaction was heat-shocked at 3-min intervals from *t* = 5 min to *t* = 14 min by transferring 20 µL from the reaction into the corresponding Tubes C–F kept at 95°C. The quenched samples (final volume = 400 µL) were then vortexed for 10 s and allowed to cool to room temperature before transferring to HPLC vials for quantitative analysis. Reactions with 50–150 µM ATP exhibited first-order enzyme kinetics at *t* = 5-, 8-, 11-, and 14-min time courses, while reactions with 200–700 µM ATP exhibited first-order enzyme kinetics at 7-min intervals from *t* = 5-, 12-, 19-, and 26-min time courses. Each reaction condition was performed three independent times. The samples were injected (40 µL) and ATP and c-di-AMP peaks were detected using a Kinetex 5 µm C18 100 Å, LC Column 150 × 4.6 mm (Phenomenex, Cat# 00F-4601-E0). Percent c-di-AMP produced from each reaction was determined based on the ratio of the peak area of c-di-AMP to the peak area of ATP. ATP and c-di-AMP signals were compared to commercial standards. A standard curve of c-di-AMP was generated to be able to convert peak areas of c-di-AMP to the concentration of c-di-AMP (µM) (Fig. S8). The HPLC method is as follows: 0–22 min (1%–13% acetonitrile/100 mM TEAA), 22–24.5 min (13%–90% acetonitrile/100 mM TEAA); 24.5–28.5 min (90% acetonitrile/100 mM TEAA); 28.5–28.51 min (90%–1% acetonitrile/100 mM TEAA); and 28.51–36 min (1% acetonitrile/100 mM TEAA). Flow rate: 0.5 mL/min. Oven temperature: 30°C. Signals were analyzed using a 254 nm UV detector.

### Intrinsic fluorescence binding assay

An intrinsic fluorescence binding assay was adopted to determine the binding dissociation constant (*K*_*d*_) of (+)-brazilin with *sm*DAC (22). Serial-diluted (+)-brazilin stock solutions in DMSO from a 5 mM master stock in DMSO were freshly prepared on the day of experiments. Samples of 400 µL containing 10% DMSO or inhibitor and 5 µM *sm*DAC in 20 mM sodium phosphate buffer pH 7.5 were incubated at 30°C for 4 h statically in a heat block protected from light. Samples without *sm*DAC were also incubated using the same conditions for background subtraction. Then, 300 µL of each sample was transferred to a 96-well plate. A Cary Eclipse Fluorescence Spectrophotometer equipped with a microplate reader accessory was used to measure the fluorescence intensity of tyrosine with excitation and emission wavelengths of 260 and 303 nm, respectively. Voltage = 900 V.

### Statistical analysis

The analyses of the *in vitro* experimental data were performed by Student’s *t*-test, one-way ANOVA, and Dunnett’s multiple comparisons test using GraphPad Prism 10.0.3. Error bars denote the SEM. Differences were significant with a value of *P* ≤ 0.05. Experiments were repeated at least in triplicate and independently.

## Data Availability

All the raw data not shown in the supplemental material are available upon request from the corresponding author H.W. (wuhu@ohsu.edu).

## SUPPLEMENTAL MATERIAL

Supplemental material spectrum.02418-23-s0001.docx Supplemental figures and tables.

ASM does not own the copyrights to Supplemental Material that may be linked to, or accessed through, an article. The authors have granted ASM a non-exclusive, world-wide license to publish the Supplemental Material files. Please contact the corresponding author directly for reuse.
