# Supplementary material for: Tetracyclic homoisoflavanoid (+)-brazilin: a natural product inhibits c-di-AMP-producing enzyme and Streptococcus mutans biofilms
Source: Microbiol Spectr. 2024 Apr 9;12(5):e02418-23. doi: 10.1128/spectrum.02418-23 (PMC11064632; doi:10.1128/spectrum.02418-23)
Supplement: Supplemental material — Supplemental figures and tables. [file spectrum.02418-23-s0001.docx]

**Tetracyclic homoisoflavanoid (+)-brazilin: A natural product inhibits c-di-AMP producing enzyme and *Streptococcus mutans* biofilms**

Edwin M. Rojas,^1,2^ Hua Zhang,^3^ Sadanandan E. Velu,^2*^ and Hui Wu^3*^

^1^School of Dentistry and ^2^Department of Chemistry, University of Alabama at Birmingham, Birmingham, AL 35294, USA; ^3^Division of Biomaterial and Biomedical Sciences, Oregon Health & Science University School of Dentistry, Portland, OR 97239, USA

Edwin M. Rojas, School of Dentistry and Department of Chemistry, University of Alabama at Birmingham, Birmingham, AL 35294, USA, Phone: (203) 942-1171, E-mail: [emrojas@uab.edu](mailto:emrojas@uab.edu)

Hua Zhang, Division of Biomaterial and Biomedical Sciences, Oregon Health & Science University School of Dentistry, Portland, OR 97239, USA, Phone: (205) 218-6502, E-mail: [zhanghu@ohsu.edu](mailto:zhanghu@ohsu.edu)

*Co-corresponding authors:

Hui Wu, Division of Biomaterial and Biomedical Sciences, Oregon Health & Science University School of Dentistry, Portland, OR 97239, USA, Phone: (503) 418-2090, E-mail: [wuhu@ohsu.edu](mailto:wuhu@ohsu.edu)

Sadanandan E. Velu, Department of Chemistry, University of Alabama at Birmingham, 901 14^th^ Street S. Birmingham, AL 35294, USA, Phone: (205) 975-2478, Email: [svelu@uab.edu](mailto:svelu@uab.edu)

**Supporting Information**

1. General Information
2. Experimental Section
3. Size exclusion chromatography and SDS page of *sm*DAC
4. HPLC chromatograms of *sm*DAC enzymatic reactions with different metal cofactors
5. Determination of optimal Mn^2+^ concentration for *sm*DAC activity
6. Screening of natural product library using the coralyne assay and using HPLC assay
7. HPLC chromatograms of *sm*DAC enzymatic reactions treated with (+)-brazilin
8. Modified Sterne-Volmer equation to determine the binding association constant (K_a_) and binding dissociation constant (K_d_)
9. Optibrium properties of (+)-brazilin and AMP
10. HPLC Calibration curve of c-di-AMP
11. References
12. **General Information**

All solutions were freshly prepared using volumetric flasks, filter-sterilized through 0.22 µm Millex-GV filters or autoclaved when appropriate, and stored at 4 °C. The desired pH for the enzymatic reaction buffers and HPLC mobile phase buffer were achieved using a SevenEasy Mettler Toledo pH meter equipped with a temperature probe. All screening compounds obtained from the NCI **(**[**https://cactus.nci.nih.gov/ncidb2.2/**](https://cactus.nci.nih.gov/ncidb2.2/)**)** were 10 mM solutions in DMSO and stored at -20 °C. Suramin hexasodium salt (Thermo Scientific, Cat # AC328540500), bromophenol thiohydantoin (ChemDiv Inc., Cat # 0168-0363), tannic acid (Thermo Scientific, Cat # AC419995000), and (+)-brazilin (MilliporeSigma, Cat # SML2132) were purchased commercially and stored at -20 °C, away from light.

The coralyne assays were performed on different days using a Cary Eclipse Fluorescence Spectrophotometer equipped with a microplate reader accessory. Coralyne chloride was synthesized from papaverine hydrochloride (MilliporeSigma, Cat # P3510), as reported in the literature, and stored in an amber vial at 4 °C for short-term and -20 °C for long-term storage, away from light (1). Concentrated stock solutions of coralyne chloride in milli-Q water were freshly prepared for each day a coralyne assay was performed. The concentration of coralyne chloride was determined by UV-Vis spectroscopy at A_420_ using Beer’s Law and the reported extinction coefficient (14,500 M^-1^cm^-1^) in water (2).

ATP (Thermo Scientific, Cat # R0441) and c-di-AMP (InvivoGen, Cat # tlrl-nacda) were purchased commercially, and aliquots were stored at -20 °C. Aliquots of 112 mg/mL *S. mutans* diadenylate cyclase (*sm*DAC) in 20 mM Tris (pH 8.0), 100 mM NaCl were stored at -80 °C. Diluted stock solutions of *sm*DAC were freshly prepared using the calculated MW = 17,750.24 Da. HPLC reactions were performed on different days using a Shimadzu HPLC with the following parts: DGU-20A3 Prominence Degasser, FCV-11AL Valve Unit, 2 x LC-20AD Prominence Liquid Chromatographs, SIL-20AC HT Prominence Auto Sampler, CBM-20A Prominence Communications Bus Module, SPD-M20A Prominence Diode Array Detector, CTO-20AC Prominence Column Oven, and Rack Changer C. Raw data from the HPLC chromatograms were exported as text files and plotted using GraphPad Prism 10.0.3.

Fluorescence microscopy experiments were performed on different days using an EVOS M5000 Imaging System at 4X magnification with scale bars of 750 µm. Dextran-cascade blue conjugated dye (10,000 MW, anionic, lysine flexible; Invitrogen by Thermo Fisher Scientific, Cat # D1976), SYTO9 green-fluorescent nucleic acid stain (Invitrogen by Thermo Fisher Scientific, Cat # S34854) and propidium iodide (Invitrogen by Thermo Fisher Scientific, Cat # P1304MP) were purchased commercially and stored at -20 °C away from light. GFP green light (brightness 0.066), RFP red light (brightness 1.447), and DAPI blue light (brightness 0.157) were the filters used for visualizing bacteria, eDNA, and glucans, respectively.

1. **Experimental Section**
2. **Size exclusion chromatography and SDS page of *sm*DAC**

**
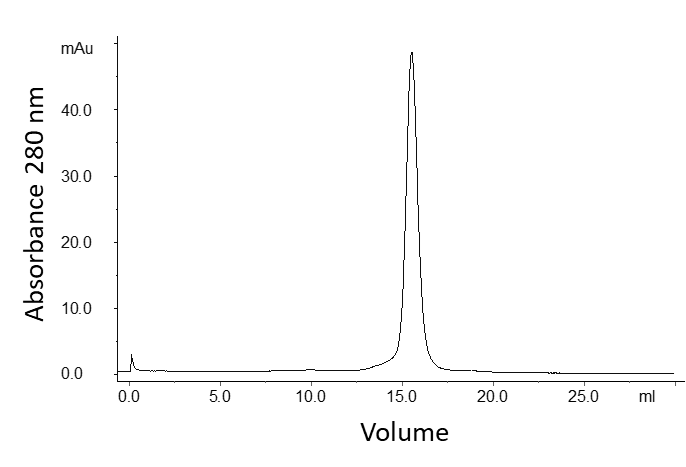

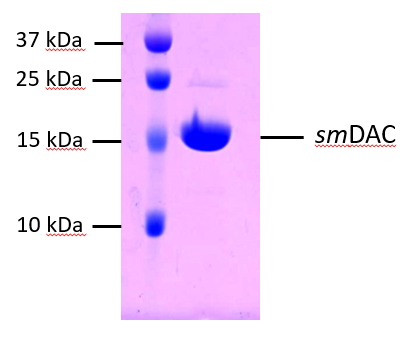
**

**Figure S1.** Size exclusive chromatography of *sm*DAC (left) and *sm*DAC on the 15% SDS-PAGE gel (right).

1. **HPLC chromatograms of DAC enzymatic reactions with different metal cofactors**

Results of the peak area and peak area % of ATP and c-di-AMP from HPLC of *sm*DAC reactions in the absence and presence of metal cofactors (CaCl_2_, MgCl_2_, CoCl_2_, and MnCl_2_).

| **10 mM metal** | **Peak Area_ATP_%** | **Peak Area_c-di-AMP_%** |
| --- | --- | --- |
| CaCl_2_ | 100 | 0 |
| MgCl_2_ | 100 | 0 |
| CoCl_2_ | 77 | 23 |
| MnCl_2_ | 19 | 81 |
| No metal | 100 | 0 |
| MnCl_2_ w/out DMSO | 23 | 77 |

**Figure S2.** Representative HPLC chromatograms of *sm*DAC reactions in the presence and absence of metal cofactors (CaCl_2_, MgCl_2_, CoCl_2_, and MnCl_2_) and summary of the results.

1. **Determination of optimal Mn^2+^ concentration for *sm*DAC activity**

**a.**

**b.** Results of the peak area and peak area % of ATP and c-di-AMP from HPLC reactions using various concentrations of MnCl_2_ (0.1, 1, 2, 5, 10, 15 and 20 mM) to determine the optimal Mn^2+^ concentration for *sm*DAC activity.

| **[MnCl_2_] (mM)** | **Peak Area_ATP_%** | **Peak Area_c-di-AMP_%** |
| --- | --- | --- |
| 0.1 | 100 | 0 |
| 1 | 75 | 25 |
| 2 | 52 | 47 |
| 5 | 21 | 79 |
| 10 | 21 | 79 |
| 15 | 27 | 73 |
| 20 | 34 | 66 |

**Figure S3.** Plot showing the % conversion of ATP to c-di-AMP using various concentrations of MnCl_2_ (0.1, 1, 2, 5, 10, 15 and 20 mM) to determine the optimal Mn^2+^ concentration for *sm*DAC activity(a) and the summary results (b).

1. **Screening of natural product library using the coralyne assay (a)and HPLC assay (b)**

**a.**

**b.**

| **NSC code** | **Peak Area_ATP_%** | **Peak Area_c-di-AMP_%** | **Normalized Enzymatic Inhibition (%)** |
| --- | --- | --- | --- |
| 70861 | 62 | 38 | 59.1 |
| 8661 | 88 | 12 | 87.1 |
| 30665 | 24 | 76 | 18.3 |
| 123262 | 81 | 19 | 79.6 |
| 113490 | 64 | 36 | 61.3 |
| 353720 | 55 | 45 | 51.6 |
| 48434 | 19 | 81 | 12.9 |
| 39882 | 40 | 60 | 35.5 |
| 59266 | 32 | 68 | 26.9 |
| 93087 | 52 | 48 | 48.4 |
| 10447 | 18 | 82 | 11.8 |

Validation of 11 “hits” at 100 µM using HPLC.

**Note:** NSC #’s 85474, 61640, and 54304 were unfortunately not evaluated using HPLC due to insufficient quantities.

**Figure S4.** Screening of a polyphenol natural products library of 20 compounds obtained from the NCI using the coralyne assay at 100 µM. Compounds with >75% reduction of fluorescence were considered “hits” and validated using HPLC.

1. **HPLC chromatograms of DAC enzymatic reactions treated with (+)-brazilin**

**Figure S5.** Representative HPLC chromatograms of *sm*DAC enzymatic reactions treated with dose-dependent concentrations of (+)-brazilin. HPLC experiments were repeated three times on different days.

| **[(+)-brazilin] (µM)** | **Peak Area_ATP_%** | **Peak Area_c-di-AMP_%** | **Normalized Mean Enzymatic Inhibition (%)** |
| --- | --- | --- | --- |
| 300 | 95 | 5 | 91.8 |
| 250 | 92 | 8 | 89.5 |
| 200 | 90 | 10 | 87.8 |
| 150 | 89 | 11 | 87.4 |
| 100 | 91 | 9 | 85.3 |
| 50 | 82 | 18 | 72.7 |
| 25 | 59 | 41 | 47.5 |
| 12.5 | 35 | 65 | 18.5 |
| 6.25 | 27 | 73 | 8.0 |
| 3.125 | 23.5 | 76.5 | 5.3 |
| 1.5625 | 21.5 | 78.5 | 3.6 |
| 0.78125 | 20 | 80 | 1.3 |
| 0.390625 | 20 | 80 | 0.8 |
| 0 | 20 | 80 | 0 |

**Table S1.** Representative Table of *sm*DAC enzymatic reactions treated with dose-dependent concentrations of (+)-brazilin. HPLC experiments were repeated three times on different days.

| **Analyte** | **Peak Area_ATP_%** | **Peak Area_c-di-AMP_%** | **Normalized Enzymatic Inhibition (%)** |
| --- | --- | --- | --- |
| 10% DMSO | 19 | 81 | 0 |
| 50 µM bromophenol thiohydantoin | 22 | 78 | 3.7% |
| 50 µM suramin hexasodium salt | 20 | 80 | 1.2% |
| 50 µM tannic acid | 37 | 63 | 22.2% |
| 50 µM (+)-brazilin | 82 | 18 | 77.8% |

**Table S2.** *sm*DAC enzymatic reactions treated with 50 µM of known *tm*DisA positive controls compared to 50 µM (+)-brazilin and the negative control reaction with 10% DMSO.

1. **Modified Sterne-Volmer equation to determine the binding association constant (K_a_) and binding dissociation constant (K_d_) (3,4)**


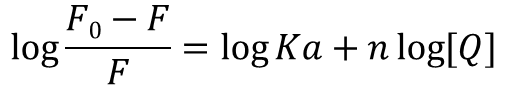
 **(Eq. S1)**

**F_0_ =** Fluorescence intensity (at 303 nm) in the absence of (+)-brazilin

**F =** Fluorescence intensity (at 303 nm) in the presence of a given concentration of (+)-brazilin

**K_a_ =** Binding association constant (M)

**n =** Predicted number of binding sites

**Q =** Given concentration of (+)-brazilin (M)

**Binding dissociation constant (K_d_) determination:**

$K_{d}= \frac{1}{K_{a}}$ **(Eq. S2)**

| **[(+)-brazilin] (µM)** | **Mean fluorescence intensity at 303 nm** | **Standard error of the mean (SEM)** | **Log[(+)-brazilin] (µM)** | **Mean log**$\frac{\boldsymbol{(}\boldsymbol{F}_{\boldsymbol{0}}\boldsymbol{-F)}}{\boldsymbol{F}}$ |
| --- | --- | --- | --- | --- |
| 100 | 50.00733667 | 3.9106 | 2 | 0.0418164 |
| 50 | 61.32907 | 5.5553 | 1.698970004 | 0.033415 |
| 25 | 79.21927 | 2.9145 | 1.397940009 | 0.024915 |
| 12.5 | 85.5903 | 3.4391 | 1.096910013 | 0.0227048 |
| 6.25 | 114.74033 | 4.1502 | 0.795880017 | 0.0154086 |
| 3.125 | 149.87255 | 6.0730 | 0.494850022 | 0.0091458 |
| 1.5625 | 162.32477 | 8.7396 | 0.193820026 | 0.0064224 |
| 0 | 173.3531467 | 5.7671 | undefined | -0.043132332 |

**Table S3. Spreadsheet showing how the Modified Sterne-Volmer plot was obtained.**

1. **Optibrium properties of (+)-brazilin and AMP**


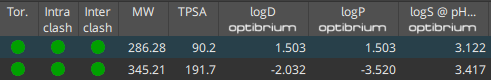


**(+)-brazilin**

**AMP**

**Figure S6.** The table generated from SeeSAR (BioSolveIT) compares the molecular weight (MW), topological polar surface area (TPSA), and Optibrium properties (LogD, LogP, and LogS at pH 7.4).

1. **HPLC Calibration curve of c-di-AMP**

**Figure S7.** A calibration curve was generated from 0, 0.5, 1, 3, 5, 8, 10, 15, and 20 µM c-di-AMP to convert peak areas of c-di-AMP product to concentrations of c-di-AMP. Experiments were performed in triplicate.

1. **References**

1. Zee-Cheng, K. Y., and Cheng, C. C. (1972) Practical Preparation of Coralyne Chloride. *Journal of Pharmaceutical Sciences* **61**, 969-971

2. Zhou, J., Sayre, D. A., Zheng, Y., Szmacinski, H., and Sintim, H. O. (2014) Unexpected Complex Formation between Coralyne and Cyclic Diadenosine Monophosphate Providing a Simple Fluorescent Turn-on Assay to Detect This Bacterial Second Messenger. *Analytical Chemistry* **86**, 2412-2420

3. Abou-Zied, O. K., and Al-Shihi, O. I. K. (2008) Characterization of Subdomain IIA Binding Site of Human Serum Albumin in its Native, Unfolded, and Refolded States Using Small Molecular Probes. *Journal of the American Chemical Society* **130**, 10793-10801

4. Opoku-Temeng, C., and Sintim, H. O. (2016) Inhibition of cyclic diadenylate cyclase, DisA, by polyphenols. *Scientific reports* **6**, 25445-25445
